# Experiences of Food and Mealtimes Among People Living in Homelessness

**DOI:** 10.1177/23333936261450410

**Published:** 2026-05-13

**Authors:** Elin Hjorth, Sandra Doveson, Anna Klarare, Viktoria Wallin

**Affiliations:** 1 Marie Cederschiöld University, Stockholm, Sweden; 2 Sophiahemmet University, Stockholm, Sweden; 3 Boston College, Chestnut Hill, MA, USA; 4 Uppsala University, Sweden

**Keywords:** food and mealtimes, homelessness, nursing, nutrition, qualitative research, Sweden

## Abstract

Food plays a vital role in daily life, not only physically but also psychologically, socially, and existentially. People experiencing homelessness often face difficulties in accessing food. This study aimed to explore experiences of food and mealtimes among people experiencing homelessness using a qualitative descriptive design. Individual interviews were conducted with 15 participants recruited from an inpatient ward specializing in care for people living in homelessness. The data were analyzed using inductive qualitative content analysis, with an emphasis on remaining close to the participants’ own descriptions. The findings provide insight into the different ways in which people experiencing homelessness manage their daily food strategies. Furthermore, how people perceive physical suffering related to hunger, but also on how support from aid organizations is experienced, and how moral boundaries may shift when the need for food becomes urgent. The intricate interplay between food insecurity, substance use, and social marginalization is explored. The study concludes that limited and unpredictable access to food has significant consequences for everyday life among people experiencing homelessness, underscoring the need for respectful and inclusive support systems within nursing practice and public policy.

## Introduction

Homelessness is a complex social phenomenon, rooted in both structural and individual factors. Substance use disorder, mental health problems and a lack of stable income are among the most frequently cited causes of homelessness in Sweden (National Board of Health and Welfare, 2024). Research shows that people living in homelessness with complex medical conditions are particularly vulnerable, as they often require various types of healthcare interventions, addressing both physical and mental health needs (Håkanson & Öhlén, 2016; Richards & Kuhn, 2023; Stajduhar et al., 2019). People experiencing homelessness have a significantly shorter life expectancy compared to those with stable housing (Aldridge et al., 2018; European Commission, n.d.; Gaber et al., 2024; Seastres et al., 2020). At the same time, people experiencing homelessness face greater barriers to accessing healthcare (Hudson et al., 2016; Stajduhar et al., 2019), since they commonly must prioritize finding shelter and food over seeking healthcare even when the need is urgent. Healthcare bureaucracy and experiencing stigma and discrimination are examples of other barriers to seeking out healthcare among people experiencing homelessness (Kneck et al., 2021). Staff working with people living in homelessness must therefore develop their skills to identify needs associated with physical illnesses (de Veer et al., 2008; Klop et al., 2018).

The human right to food and nutrition has been upheld by the United Nations (2025) for more than half a century, nonetheless, people living in homelessness often have limited access to food, and difficulties in preparing and storing food as well as eating. Living in homelessness presents numerous barriers to satisfying hunger, thirst, and basic nutritional needs (Seale et al., 2016; Sprake et al., 2014). Food is essential not only for survival but also for structuring daily life and social interactions (Sutton, 2010). Mealtimes often involve human connection, and preparing and eating together can strengthen relationships (Jastran et al., 2009; Nettleton & Uprichard, 2011) and contribute to one’s well-being and sense of security (Louge, 2004). However, severe illnesses can disrupt these interactions, as eating difficulties reduce opportunities to share meaningful moments with loved ones (Del Rio et al., 2012; Wallin et al., 2021). Further, eating difficulties can be an existentially charged marker of illness severity and frailty of life (de Veer et al., 2008; Klop et al., 2018). Compared with the general population, people experiencing homelessness more often report poor oral health (Watt et al., 2019), with pain that may interfere with eating (Freitas et al., 2019). Substance use disorder commonly exacerbates the problem by negatively affecting both food intake and nutrient absorption (Tse & Tarasuk, 2008). Physical illnesses, which are common in people experiencing homelessness, also affect appetite, nutrient intake, and -absorption (Preedy, 2011; Tan & Fearon, 2008). There is a clear association between uncertain access to food and multimorbidity. However, the causal relationship remains unclear, as a recent meta-analysis review also indicates a reverse association between uncertain access to food and multimorbidity (Kantilafti et al., 2023).

In summary, the literature shows that food and mealtimes play a vital role in daily life, including physical, psychologically, social and existential dimensions. However, few studies focus on experiences of food and mealtimes among people living in homelessness. Understanding these perspectives can elaborate how homelessness affects basic human needs, and how access to food affects other aspects of life. The aim of this study was therefore to explore experiences of food and mealtimes among people living in homelessness.

## Methods

### Study Design

This study used a qualitative descriptive design and individual interviews with people experiencing homelessness. The approach emphasized staying close to the data and participants’ own words to produce an accurate and meaningful account of their experiences (Sandelowski, 2000). Data were analyzed using inductive qualitative content analysis (Graneheim & Lundman, 2004).

### Context

In Sweden, homelessness has various forms, such as temporary, insecure, or institution-based housing and being limited to street homelessness. Within the Swedish welfare system, municipalities have primary responsibility for social services, including support for people experiencing homelessness. This means that access to housing support and basic services may vary locally. Within this context, municipal social services provide emergency and temporary accommodation, ranging from overnight shelters to short- and medium-term housing solutions with around-the-clock staffing. These housing arrangements are typically conditional, time-limited, and dependent on availability. In these facilities, residents are usually provided with three mealtimes per day as well as access to snacks, with meals served at set times and limited opportunities for individual choice or participation in food preparation. In parallel with municipal services, a substantial part of food provision for people experiencing homelessness is organized by non-profit, volunteer-based organizations, both secular and faith-based. These actors distribute food either at street level, for example through soup kitchens, or in connection with shelters and open meeting places. Access to these services is generally low-threshold but is also constrained by opening hours, capacity limitations, and reliance on donations.

Participants in the present study were experiencing homelessness within this Swedish welfare context. At the time of data collection, all participants were temporarily admitted to an inpatient ward.

### Participants and Recruitment

Participants were recruited from an inpatient ward in an urban Swedish city that specializes in the care of individuals experiencing homelessness. The ward provides both urgent and planned care, including both medical treatment, nursing care and rehabilitation. The ward is specialized in complex needs related to circumstances surrounding physical and mental illness, including substance use disorder. The ward also collaborates closely with social services and primary care. Recruitment was conducted by three registered nurses, appointed by the ward manager, who informed eligible individuals about the study and invited them to participate. The nurses were informed about the study and maintained direct contact with the interviewing researcher, who was present at the ward several days per week during the data collection period.

Eligible participants were receiving care in a specialized ward for individuals experiencing homelessness and met the criteria for current homelessness according to the definition by the European Federation of National Organisations Working with the Homeless (FEANTSA, 2005). This definition identifies four main categories that together capture both visible and “hidden” forms of homelessness: rooflessness, houselessness, insecure housing, and inadequate housing. The length of time a person had experienced homelessness was not decisive. Both men and women were included, and participants were required to be able to converse in Swedish or English.

Participation was voluntary and their care was not affected by the decision to participate in the study or not. Interviews were conducted at the ward, in either a private conference room or in the participant’s patient room. The Regional Ethical Review Board (now the Swedish Ethical Review Authority) approved the study prior to its commencement (no. 2019-02454).

### Data Generation

An interview guide was developed drawing on previous research on food, meals, and homelessness. Open-ended questions such as “*Can you please describe how mealtimes are for you when you do not have a home on your own?*,” “*What does a typical day of eating look like, can you describe?*,” “*How do you get food?*” were asked during the qualitative interviews, with follow-up probes for more in-depth responses (Price, 2002). Individual interviews were conducted. Three of the participants were, upon their own request, interviewed twice, resulting in a total of 18 interviews. All interviews were carried out by VW. The interviews were audio-recorded with participant consent, except for two cases where participants preferred not to be recorded but wished to share their experiences. In these cases, detailed written notes were taken during and after the interviews.

### Data Analysis

An inductive qualitative content analysis, as described by (Graneheim & Lundman, 2004) and further elaborated by Lindgren et al. (2020) was conducted. The interviews were transcribed verbatim, and the transcripts were read multiple times to gain a comprehensive understanding. The analysis was primarily conducted by EH and SD in collaboration with AK and VW. Meaning units were highlighted and extracted from the transcripts. A meaning unit constitutes a segment of text (ranging in length from a single sentence to several sentences) that pertains to the study aim. All meaning units were then condensed, thereby shortened to a more manageable length. Each condensed meaning unit was subsequently assigned a code, which served as a label capturing the essence of the text in a few words. The codes were used as operators to search for similarities and differences between the text segments, all while moving back and forth between the de-contextualized codes and their original context in the interview. In the re-contextualizing process, the codes were used to form themes and subthemes based on similarities and differences. Interviews from all participants contributed to the findings and selected quotations were used to illustrate them. The findings were repeatedly read, reflected upon, and discussed among all authors as part of the inductive qualitative content analysis process until consensus was achieved.

## Findings

The findings are presented through three themes, *Everyday struggling with hunger and physical well-being*, *Receiving help and trying to preserve dignity*, and *Crossing one’s own moral boundaries trying to survive*. Together they capture how food and mealtimes are experienced as central and challenging aspects of daily life for people facing homelessness. They show that eating is not just about nutrition but involves ongoing struggles and compromises, illustrating the complex social, existential and physical realities surrounding food in this context.

### Description of the Participant Group

Demographic data, including age, time spent in homelessness, occupation, and reasons for being on the ward, were self-reported. Fifteen participants participated in the study (4 women and 11 men). The participants’ ages ranged from approximately 30 to 73 years, two were not sure about their age and therefore self-estimated their age. Most had experienced homelessness for several years, with durations varying from a few months to up to 18 years. Some found it difficult to specify exact timelines due to changes over the years. Most of the participants described themselves being so called “rough sleepers,” in other words, sleeping on buses or stairwells, in temporary shelters and during summer maybe in an outdoor setting. Two participants did not consider themselves homeless, as they usually stayed with friends.

The participants’ time on the hospital ward ranged from 3 days to 3 weeks, and they had been referred there either from different acute hospital wards or from a health center for marginalized groups. Care needs were related to infected wounds on feet or legs, burns, memory loss, epilepsy, or chronic conditions like chronic obstructive pulmonary disease, HIV, and cancer. While some participants described these conditions as well-managed, others reported lapses in treatment or follow-up. Substance- and alcohol use were commonly described by the participants, even though the interviewer did not directly ask about it, and most participants openly discussed their own or others’ struggles with addiction. A few participants reported no issues with substance use. The participants had diverse educational and occupational backgrounds. While some had not completed primary school, three had attended university. Participants described having worked in various roles, such as technicians, fitters, engineers, cleaners, window cleaners, cooks, kitchen assistants, and bar staff. Two participants had previously run their own small businesses, two were currently employed, and others occasionally took on temporary work or volunteered at treatment centers. The reliability of some background information was limited, as some were uncertain about their age, diagnoses, or the severity of their conditions.

### Everyday Struggling With Hunger and Physical Well-Being

The participants’ relationship to food was described in terms of its importance for physical health and survival, framed by the context of their difficult living conditions. Participants also described the experience of hunger, which was portrayed as a deeply distressing state affecting both body and mind. Hunger could alter thoughts, emotions, and behaviors, and was said to lead to aggression, paranoia, and in some cases mimic symptoms of psychosis or drug use. One participant expressed:

Food means everything, you know. It’s energy, it’s what keeps your head straight and your body, like, all your organs working. If you don’t get food and sleep, lots of functions just shut down, and if it’s food you’re missing you get aggressive, even paranoid, you know? So when you see a lot of homeless people talking to themselves and acting angry, it’s not necessarily drugs—it’s just that they haven’t eaten. (Participant 9)

Food was seen as having a central role in rebuilding and strengthening the body, particularly during illness or after injury. Participants showed awareness of the importance of eating a varied diet, and some tried to supplement their intake with vitamins. Prolonged hunger also often led participants to overeat once they had access to food. One participant described how the body could react paradoxically after prolonged hunger, once food was available, the body could start vomiting instead of absorbing the nutrients. During periods when participants prioritized alcohol and drugs over food, they still described attempts to care for themselves—for example, by at least drinking water as a minimum. Nutritional deficiencies were perceived to affect both health and energy levels, and a lack of nutritious food was linked to hair loss, liver damage, and poorer overall health by the participants. Furthermore, the participants communicated gratitude for having access to food through voluntary organizations and food distribution centers. At the same time, they voiced frustration regarding that the food provided often lacked enough nutritional value and mostly consisted of bread and sweet pastries. One participant described:

If you go to all the distributions, you never really have to go hungry, so to speak. It’s enough. Yeah, the only thing is you get really tired of sweet pastries—there’s loads of that. It’s probably not the healthiest either, but still. . . it takes the edge off the hunger, at least a little. (Participant 12)

Despite this awareness, several participants described how substances were sometimes prioritized over food. Substances were described not only as a way to quell hunger, but also as a necessary escape from the harsh reality of homelessness. For participants with substance use experiences, it was the substances, rather than the food, that were at the center of their daily thoughts and actions. One participant explained that substances provided a temporary escape and helped to reduce both physical and mental sensations of hunger. The direct effect of the substances was to further reduce the importance of food. Alcohol was also mentioned as a substitute for food. Participants described using beer to relieve hunger pangs and fill their stomachs, while other substances were reported to temporarily ease the situation:

As an addict, you don’t really think about food. You think about drugs. Those times I’ve wandered around at night. . . well, you take a bit of amphetamine, you know, to. . . yeah, it warms you up, gives you the energy to stay awake. I can’t speak for others, but it’s almost like you have to take something just to numb things a bit, like an escape from reality, just to cope. Of course it affects your appetite. [. . .] But if you take some amphetamine, it kills your appetite, so it kind of goes hand in hand, you know? You’re freezing and you’re hungry, and you’ve got a hamburger over here and some amphetamine over there—I’m speaking from my own experience—but 99% of the time, it’s the amphetamine you go for. Because it kills the hunger and gives you that escape. The hamburger just makes you full, but that escape. . . that sticks in your head, you know? (Participant 2)

### Receiving Help and Trying to Preserve Dignity

Participants experienced that various organizations and volunteer groups played a central role in providing access to food. These organizations were a lifeline for many, though not without criticism. Some participants reported feeling “infantilized,” for instance, when sandwiches were prepared for them, and food was handed out with an air of dismissiveness. Participants described being treated like children or individual’s incapable of taking care of themselves. At the same time, they expressed a clear awareness that this help was essential, and without it, survival and everyday life would be much more difficult. Participants described strategies for navigating between different distribution points to maximize their chances of obtaining food throughout the day. In some cases however, food distribution was perceived as conditional. Requirements varied between organizations, with some incorporating religious elements into their services. For instance, to receive something like a sandwich, people might be expected to attend a religious meeting or read religious material—conditions that were experienced as problematic, especially for those who did not share those beliefs:

To get a sandwich there, you have to join their morning meeting and read about God. And if you’re not religious or Christian, well, then you don’t get any food. I mean, it’s like forcing people to. . . so that we can feel good and make it look good. It’s really messed up, because then you’re not giving out of love—you’re giving to get recognition. And there’s a big difference between giving with no strings attached. (Participant 9)

Additionally, the food distributed was often not suited to the specific needs of people living in homelessness. One example was the distribution of canned beans, even though many lacked can openers or the means to cook the food. Another example was hard cracker bread, which was difficult for participants without teeth or with chewing difficulties to eat.

The participants shared how they had received help from strangers who offered food and beverages in difficult situations. One participant described a period of living in a gazebo without walls, where passers-by, noticing the situation, approached and offered food and water. The participant expressed deep gratitude for these gestures and described a form of “karma,” giving and receiving in return. Another participant noted an increase in food donations during December, when people tend to be more generous—an act the participant interpreted as an expression of guilt. Occasionally, people bought food from restaurants to distribute, but such initiatives were described as relatively rare outside the holiday season.

Some restaurants and stores offered food that would otherwise have been thrown away. One participant shared often going into pizzerias to explain the situation in hopes of getting food. Certain stores gave away items past their expiration date, which were described as a valuable resource by the participants. However, they also experienced stigmatization and distrust from both the public and restaurant owners. They described instances of being denied entry because they were perceived as “dirty” or suspected of having illnesses. One participant described:

You look different after living on the streets, and when you ask someone (for food), the first reaction you get is that they’re scared. It’s really hard to go up to someone yourself, you know, to ask. But later you have no choice—when your body really demands it, when it’s been a day without water or food, then you’re forced to. Maybe it’s the survival instinct taking over and giving you that push. [. . .] Then it’s about either working up the courage or starving. (Participant 5)

Despite their own hardships, participants described other homeless persons as generous. They noted that it was common to share food with others in equal need, fostering a sense of community and solidarity among people experiencing homelessness. This generosity was especially evident in situations where food was scarce and access uncertain. One participant shared how they often waited until the end of a food distribution event to see if anything was left over, so it could be shared with others. Another described a situation where they collected money so that someone who was ill could buy nutritional supplements at the local pharmacy. The sharing of food was also balanced against a sense of needing to prioritize oneself when resources were scarce or lacking, a sort of “every man for himself” situation, as described by one participant:

Of everyone I’ve met, it’s probably homeless people who share the most. You help each other out, and if someone’s got a meal, someone else comes up and shares it, gives you something so you’ve got something too—so both of you have something. But at the same time, it’s also like, no one has anything and everyone’s just trying to save themselves. Everyone’s just trying to survive. So yeah, I got robbed, all my stuff was gone, that kind of thing happened too. . . all the time. Like, yeah, everyone’s just trying to find a way to survive. (Participant 5)

### Crossing One’s Own Moral Boundaries Trying to Survive

The participants’ narratives reflect how perceptions of food, begging, theft, and moral boundaries shifted over time in the struggle for survival. In moments of desperation, they described crossing social norms and taboos in their search for food. A common strategy for obtaining food was collecting leftovers from public places, such as fast-food restaurants—something they had never imagined themselves doing before experiencing homelessness.

The participants described various strategies for managing food and mealtimes in daily life. Food planning was not only about meeting nutritional needs but also about survival, requiring careful consideration and constant adaptation to the uncertainty of where the next meal would come from. A recurring strategy was simply to “figure it out somehow,” meaning they would wait until they found something to eat if no food was immediately available. For some, asking for or accepting food initially evoked feelings of shame. Participants struggled with their sense of pride and principles, stating they never thought they would resort to begging. However, hunger and the need for food forced them to reevaluate previous convictions and accept help they had previously rejected. When the body truly demands sustenance, when an entire day has passed without food or water, there is no alternative. For some, asking for food gradually became a routine part of daily life. It became evident that asking for help often required considerable courage: “Having to ask for food feels fucking degrading, it really fucking hurts to feel like you can’t even afford to eat” (Participant 9).

Food scarcity could also lead to competition, with long queues in food services and the risk of food running out before everyone was served. While food had the potential to foster social interaction, the context of homelessness often limited this. Some participants described avoiding socializing at distribution centers to escape judgmental glances:

If you go to one of those food distribution places, it’s not social. You just hide from people and then leave. You’re only there for the food, because at the same time. . . people are so judgmental, you know. [. . .] Yeah, you kind of hide in the line, so it’s like. . . Yeah, for me it’s. . . I always speak from my own experience. I don’t put energy into others, so that’s how it is. (Participant 4)

Stealing food also emerged as a, often necessary, strategy. One participant described theft as a behavior driven purely by the instinct to survive. Several emphasized that stealing was not about personal gain, but about survival. Another noted that there was an “unwritten rule” among some persons living in homelessness against stealing, even in the face of severe hunger. However, this rule was occasionally broken when all other options had been exhausted.

A lot of people live completely outside of society and manage on their own, so they steal. But I’ve never stolen to sell, I’ve only stolen to eat. . . because, well, it happens. Food, clothes, or hair ties. I’ve taken what I needed just to survive. But I don’t want to keep doing that either—I’m really trying to rethink things. (Participant 14)

## Discussion

The participants’ accounts illustrate the daily struggle to manage hunger and maintain physical well-being amid profound uncertainty. These narratives highlight the tension between receiving essential help and preserving a sense of dignity, as support often came with conditions or attitudes that felt disempowering. At the same time, survival sometimes required crossing moral boundaries, whether through strategies to maximize food access or navigating situations that challenged personal values, underscoring the complexity of life during times of homelessness. While the participants recognized the importance of nutritious food to navigate their challenging lives, they faced persistent barriers related to access, resources, and health issues.

The results showed that conditional premises for food were a part of the homeless peoples’ everyday lives. In a welfare society, such as Sweden, access to food might appear to be a given. According to the United Nations (2025) food is a fundamental human right, yet the findings of this study show that this is not always the case in practice. The results show that for people experiencing homelessness, access to food is conditional and shaped by power relations. For those with substance use disorder, substances or alcohol often become the priority, pushing food to a secondary concern. In such cases, substances can serve both as strategies for enduring an extreme existence, and to dull the hunger. The results show how the perception of what is socially acceptable can shift over time. Behaviors such as begging for food, stealing, or scavenging from garbage bins emerge when survival becomes the overriding priority. This process can be described as being forced to redraw one’s own moral map, where actions that were previously considered wrong or shameful are reinterpreted in light of life-threatening scarcity. From an ethical perspective, the results can be interpreted through Nussbaum’s capability theory, where actions are understood as consequences of structural vulnerability rather than as moral deficiencies (Nussbaum, 2011). According to Nussbaum, states have a responsibility to ensure the conditions necessary for life and the realization of basic capabilities, including physical health, bodily integrity, and social participation. If a capability belongs on this list, Nussbaum argues, governments have an obligation to protect and secure it through legislation and public policy. When these conditions are unmet, people may find themselves in situations where their agency—the capacity to act and make decisions based on their own values, is constrained. The capability approach can be understood as a framework for theorizing fundamental social justice. According to Nussbaum (2011), the role of the state is not primarily to provide food for everyone, but to create the conditions that enable people to make life choices allowing them to meet their basic needs. Through Nussbaum’s lens, the individual is linked to a broader social context that may have failed them; rewriting one’s moral map can therefore be seen as a renegotiation of one’s place within a society that has already consigned one to its margins.

The importance of viewing food and mealtimes from a holistic perspective of health was evident in participants’ narratives. According to the World Health Organization’s (WHO) classical definition of health from 1948, health is not merely the absence of disease but “a state of complete physical, mental and social well-being” (World Health Organization, 1948). In line with this definition, the results do not only show the most fundamental and explicit dimension—the physical need for nutrition—but also emphasized the social and psychological significance for food and mealtimes at stake. The role of food and mealtimes extends beyond alleviating hunger: it can foster social connectedness, restore a sense of normality, and contribute to experiences of dignity and belonging. Plage et al. (2025) showed that for people experiencing homelessness access to food is a daily struggle that simultaneously (re)produces their subordinate social status. Centering health, it is crucial to promote access to food not solely to prevent nutritional deficiencies and disease, but also as a strategy to strengthen social relationships and mental well-being. This is particularly important for people experiencing homelessness, for whom food and mealtims may serve as a resource in processes of healing and recovery.

Health care professionals, especially registered nurses, may play an important role in fostering trust and thereby promoting health-seeking behaviors among people living in homelessness (Becker & Foli, 2022). A recent review explored how and why multicomponent outreach health-care services work for people experiencing exclusion (Johnson et al., 2025). The study highlights the importance of flexible care, such as tailoring appointment length to meet service users’ needs, which fosters respect and more comprehensive care. Meeting people where they live was shown to build understanding and trust. The review also emphasizes the value of addressing immediate, basic needs such as food, clothing, or shelter. By meeting these needs, services help users feel valued and cared for, strengthen relationships with staff, and create opportunities to engage with their health.

In general care, registered nurses carry a responsibility for addressing nutritional needs during care. However, a gap may arise when the person lacks stable housing and financial resources, creating a particularly challenging situation. As one of the largest healthcare disciplines, nurses are well positioned to facilitate access to care for people experiencing homelessness, as highlighted in a scoping review of nurse-led services (McWilliams et al., 2022). Their scope of practice is holistic, encompassing physical and mental health assessments, wound care, care coordination, health promotion, and education. However, the review did not identify specific nursing responsibilities regarding nutritional assessment in this population. There are, however, guidelines addressing nurses’ involvement in food, nutrition, and homelessness (The Queen’s Nursing Institute, 2020). The literature indicates that registered nurses can and should have a role in nutritional assessment and support regarding food and nutrition for people experiencing homelessness, particularly based on guidelines and studies on barriers and nutritional needs. Registered nurses could play a central role in reclaiming nutrition as part of their professional domain and, through their expertise, contribute to placing nutrition higher on the healthcare agenda especially when caring for people living in homelessness. Still, there is a lack of empirical research that clearly demonstrates the nursing role in relation to nutrition in this population, and interventions that are effective. This nursing responsibility could extend beyond assessing nutritional status and ensuring adequate intake; to also involve health promotion through knowledge dissemination and collaboration with social services, non-governmental organizations, and other stakeholders. Participants in this study described that the food distributed or otherwise available to them did not always meet their nutritional needs. Previous research supports this, showing that factors such as financial instability and the type of food provided by aid organizations can contribute to malnutrition among people experiencing homelessness (Seale et al., 2016; Sprake et al., 2014). Crawford et al. (2015) showed that young persons living in homelessness supported by specialized homelessness services did not reach the recommended minimum levels of basic foods such as vegetables, bread and cereals. At the same time, their consumption of sugar-sweetened beverages was high. However, some studies highlight exceptions, where aid organizations deliver nutrient-rich food to individuals in low-income households (Nogueira et al., 2021). These results, together with the findings of the current study, point to a need to re-evaluate health- and social care services to improve access to quality and nutritious food. Food insecurity is a complex problem that requires a multifaceted and multidisciplinary approach to ensure better access to food and improved diet quality for persons experiencing homelessness with existing examples of how this can be conducted (Wetherill et al., 2023). Although local initiatives such as food distributions and food banks have positive effects for the people they reach, a review of interventions to address household food insecurity in high-income countries has shown that these local efforts lack evidence of effectively reducing food insecurity at the population level. The study instead suggests public policy strategies in the form of social protection to reach larger segments of the population (Loopstra, 2018). Here, registered nurses can serve as a resource and contribute to developing strategies, as well as identifying people in need.

### Implications for Nursing

Experiencing hunger is a prevailing and transformative experience that affects both body and mind. To meet the needs of people living in homelessness, health- and social care services have a joint responsibility to recognize the profound effects of hunger and consider integrating food support into interactions with vulnerable people.

The findings indicate that nurses are well positioned to contribute to developing and implementing strategies to improve access to nutritious food for people experiencing homelessness. However, the feasibility of such engagement depends on contextual factors, including workload, political and organizational priorities, and available resources. To support this expanded role, organizational changes may be required, such as integrating screening for food insecurity into routine nursing assessments at the point of hospital discharge and establishing clear pathways for collaboration with social services. Additionally, nurses may benefit from increased training and support to address hunger as part of holistic care.

Furthermore, the nursing profession could play an important role in highlighting structural needs, advocating for social protection measures. However, addressing hunger should not rely solely on individual initiatives but requires interprofessional collaboration and supportive policies to improve the health and living conditions for people living in homelessness.

### Strengths and Limitations

One of the strengths of this study lies in its perspective, which is grounded in the voices and experiences of people living in homelessness. Their own accounts are brought forward in relation to food and mealtimes, an issue that, to our knowledge, has received limited scholarly attention. The participants were recruited from a hospital ward, which may have influenced the findings due to their circumstances as inpatients. Nevertheless, given that physical illness is common among people experiencing homelessness (Håkanson & Öhlén, 2016; Seastres et al., 2020; Stajduhar et al., 2019), the results may be regarded as transferable to similar populations of people experiencing homelessness. Since epidemiological background data was not collected, some information about the participants is missing. At the same time, participants had the opportunity to describe their situation in their own words, which meant that they shared the background information they considered important for the researchers. We have sought to strengthen the rigor of the study by maintaining a systematic approach throughout the entire research process, from data collection to analysis and the reporting of results. The analysis was conducted mainly by [EH] with assistance from [SD], and multiple reviews and feedback from the co‑authors strengthened both the credibility and confirmability of the study. The transferability of the study to other countries and contexts must be assessed by the reader; however, our intention is that the detailed description of the study execution and context enables such an assessment (Flanagan & Beck, 2025).

## Conclusion

The participants’ narratives highlight food as essential, not only for physical survival but also for mental stability and social dignity. While voluntary organizations provided crucial support, the food was often nutritionally inadequate and sometimes distributed under conditions that compromised autonomy. Despite these challenges, a sense of solidarity existed within the homeless community, marked by mutual aid and resource sharing. The findings reveal complex power relations between uncertain access to food, substance use, and social marginalization, underscoring the need for more respectful and inclusive support systems. This study is relevant for public policy as it highlights how uncertain access to food affects people experiencing homelessness. The results suggest that policies and programs are needed to provide access to nutritious food for people living in homelessness. These programs must be coordinated, preserve individuals’ autonomy, and be robustly evaluated to ensure ongoing development and sustainability.
